# Medicare Annual Wellness Visit for Nursing Home Residents

**DOI:** 10.1093/geroni/igaa057.593

**Published:** 2020-12-16

**Authors:** Theodore Malmstrom, Milta Little, Angela Sanford, Marla Berg-Weger, John Morley

**Affiliations:** 1 Saint Louis University School of Medicine, Saint Louis, Missouri, United States; 2 Duke University School of Medicine, Durham, New York, United States; 3 Saint Louis University, St. Louis, Missouri, United States

## Abstract

The 2010 Patient Protection and Affordable Care Act includes the Annual Wellness Visit (AWV) for older adult (OA) patients. Medicare pays for an initial AWV per beneficiary and subsequent visits annually. Many Medicare beneficiaries have not taken advantage of the AWV preventive health benefit. The Saint Louis University Geriatrics Workforce Enhancement Program (GWEP) developed an AWV for OA, NH residents. This project describes the NH AWV and reports results. Data include age, gender, comorbidities, medications, hospitalizations, depression, frailty, pain, sarcopenia, sensory impairment, cognition, nutrition, smoking, falls, and advance directives. Two suburban academic for-profit NHs are included in this study (2016-17). OA NH residents (N=247) completed an AWV and 36.8% (n=91) had a 1-year follow-up AWV. OA NH residents were female (n = 177, 71.7%) and a majority ages 75+ (n = 172, 69.7%). Most (96.3%) had a documented advance directive. Comorbidities (7.8±2), polypharmacy (92.3%), vision impairment (52.8%), hearing impairment (52.8%), depression (65.2%), frailty (75.7%), sarcopenia (84.4%), risk of weight loss (53.9%), MCI (11.7%), and dementia (75.8%) were prevalent. Among OA NH residents (n=91) with an AWV follow-up, there was modest worsening in total comorbidities and medications as well as frailty, sarcopenia, and cognition scores (ps≤0.05). Pain, depression, and nutrition did not change. To our knowledge, no one has specifically analyzed the Medicare AWV in NHs. Data from the traditional AWV is an extension of the routine clinical care of OAs and therefore could also be useful for healthcare professionals focused on providing care to OA patients in the NH setting.

